# Comparative study of endoscopic surveillance in hereditary diffuse gastric cancer according to *CDH1* mutation status

**DOI:** 10.1016/j.gie.2017.06.028

**Published:** 2018-02

**Authors:** Emma Z. Mi, Ella Z. Mi, Massimiliano di Pietro, Maria O’Donovan, Richard H. Hardwick, Susan Richardson, Hisham Ziauddeen, Paul C. Fletcher, Carlos Caldas, Marc Tischkowitz, Krish Ragunath, Rebecca C. Fitzgerald

**Affiliations:** 1 School of Clinical Medicine, University of Cambridge, Cambridge, UK; 2 MRC Cancer Unit, Hutchison-MRC Research Centre, University of Cambridge, Cambridge, UK; 3 Department of Psychiatry, University of Cambridge, Cambridge, UK; 4 Department of Histopathology, Cambridge University Hospitals NHS Trust and University of Cambridge, Cambridge, UK; 5 Department of Oesophago-Gastric Surgery, Cambridge University Hospitals NHS Trust, Cambridge, UK; 6 Familial Gastric Cancer Study, Department of Oncology, Cambridge University Hospitals NHS Trust and University of Cambridge, Cambridge, UK; 7 Cambridge and Peterborough NHS Foundation Trust, Cambridge, UK; 8 Cancer Research UK Cambridge Institute, University of Cambridge and Cambridge University Hospitals NHS Foundation Trust, Cambridge, UK; 9 Department of Medical Genetics, University of Cambridge and Cambridge University Hospitals NHS Trust, Cambridge, UK; 10 NIHR Nottingham Digestive Diseases Biomedical Research Unit, University of Nottingham and Nottingham University Hospitals NHS Trust, Nottingham, UK

**Keywords:** AEI, allelic expression imbalance, *CDH1*+, *CDH1* pathogenic variant, *CDH1*-NPVD, *CDH1* no pathogenic variant detected, DGC, diffuse gastric cancer, EORTC-QOL-C30, European Organization for Research and Treatment of Cancer Quality of Life Questionnaire Core 30, GC, gastric cancer, HDGC, hereditary diffuse gastric cancer, PPI, proton pump inhibitor, QoL, quality of life, RRTG, risk-reducing total gastrectomy, SF-36, 36-item Short Form Health Survey, SRCC, signet ring cell carcinoma

## Abstract

**Background and Aims:**

Hereditary diffuse gastric cancer (HDGC) accounts for 1% of gastric cancer cases. For patients with a germline *CDH1* mutation, risk-reducing gastrectomy is recommended. However, for those delaying surgery or for families with no causative mutation identified, regular endoscopy is advised. This study aimed to determine the yield of signet ring cell carcinoma (SRCC) foci in individuals with a *CDH1* pathogenic variant compared with those without and how this varies with successive endoscopies.

**Methods:**

Patients fulfilling HDGC criteria were recruited to a prospective longitudinal cohort study. Endoscopy was performed according to a strict protocol with visual inspection followed by focal lesion and random biopsy sampling to detect foci of SRCC. Survival analysis determined progression to finding of SRCC according to *CDH1* mutation status. The European Organization for Research and Treatment of Cancer Quality of Life Questionnaire Core 30 and 36-item Short Form Health Survey questionnaires assessed quality of life before surveillance and each endoscopy.

**Results:**

Eighty-five individuals fulfilling HDGC criteria underwent 201 endoscopies; 54 (63.5%) tested positive for *CDH1* mutation. SRCC yield was 61.1% in *CDH1* mutation carriers compared with 9.7% in noncarriers, and mutation-positive patients had a 10-fold risk of SRCC on endoscopy compared with those with no mutation detected (*P* < .0005). Yield of SRCC decreased substantially with subsequent endoscopies. Surveillance was associated with improved psychological health.

**Conclusions:**

SRCC foci are prevalent in *CDH1* mutation carriers and can be detected at endoscopy using a standardized, multiple biopsy sampling protocol. Decreasing yield over time suggests that the frequency of endoscopy might be reduced. For patients with no *CDH1* pathogenic variant detected, the cost-to-benefit ratio needs to be assessed in view of the low yield.

Gastric cancer (GC) is the second most common cause of cancer-associated death worldwide, responsible for over 700,000 deaths annually.^1^, ^2^, ^3^ Approximately 1% to 3% of GC cases arise as a result of inherited cancer predisposition syndromes, which include hereditary diffuse GC (HDGC).^4^, ^5^ Clinical criteria for the diagnosis of HDGC (considering first- and second-degree relatives) are 2 cases of GC in the family regardless of age, with at least 1 confirmed diffuse type (DGC); 1 case of DGC under age 40 years; or a personal or family history of DGC and lobular breast cancer, 1 diagnosed below age 50 years.^6^ In families fulfilling the criteria, 25% to 50% have a germline *CDH1* mutation,^6^, ^7^, ^8^, ^9^ although rates are lower (8%-15%) in regions of high incidence of sporadic GC, such as South Korea and Japan.^10^, ^11^ The penetrance of *CDH1* mutation is high, with an estimated lifetime risk of DGC of 70% in men and 56% in women and 42% lifetime risk of lobular breast cancer in women.^6^, ^7^, ^12^ Recently, mutations in *CTNNA1* and *MAP3K6* have also been associated with HDGC.^12^, ^13^, ^14^ Other candidates are *BRCA2*, *PALB2*, *INSR*, *FBXO24,* and *DOT1L*, although the latter 3 have only been described in single families.^12^, ^15^ The significance of these mutations is uncertain as other studies of *CDH1* mutation–negative families failed to identify them and sample sizes are insufficient to determine their prevalence and penetrance.^6^, ^16^ Hence, clinical genetic testing is currently restricted to *CDH1.* In over 60% of HDGC cases, no causative mutation is identified, so patients remain uncertain about their individual risk.

Individuals found to carry a pathogenic *CDH1* variant (*CDH1*+) are advised to undergo risk-reducing total gastrectomy (RRTG) because symptomatic GC has a poor prognosis.^6^ However, some patients prefer to delay gastrectomy for medical or psychosocial reasons, for example, concerns about childbearing, impact on work, fear of surgical adverse events, or comorbidities that increase the risks of surgery.^17^, ^18^ For these patients, endoscopic surveillance is recommended to provide further evidence to help in decision-making processes. Endoscopy with biopsy sampling aims to detect microscopic foci of intramucosal signet ring cell carcinoma (SRCC) and its precursor lesions, which are characteristic of early HDGC. For HDGC families with no identified genetic cause the uncertain risk precludes RRTG, and endoscopic screening is offered as the only means to determine whether they are at risk.^6^ However, although there is some evidence for successful detection of SRCC in *CDH1* mutation carriers,^17^, ^19^ the SRCC yield in individuals from families with no confirmed mutation is unknown so the value of repeated endoscopy is uncertain. This is particularly relevant given the psychological morbidity reported in some patients undergoing regular endoscopy.^18^

The aim of this study was to assess the value of endoscopic surveillance for HDGC patients in 2 groups: (1) patients with *CDH1*+ choosing to delay gastrectomy and (2) patients with no pathogenic variant detected in their family pedigree (*CDH1*-NPVD). The primary outcome was the yield of SRCC foci on endoscopy, and the secondary outcome was the change in yield with time on endoscopic surveillance.

## Methods

### Patients

In this prospective longitudinal cohort study, patients were recruited from the Cancer Research UK Familial Gastric Cancer Registry maintained at Cambridge University Hospitals NHS Trust (MREC 97/5/32, date of approval March 30, 1998) between September 2007 and December 2016 and were included if they met the criteria for HDGC. All participants gave written informed consent. Patients were managed by a multidisciplinary team with expertise in cancer genetics, gastroenterology, gastric surgery, pathology, and psychosocial support and nutrition. All patients were offered genetic counseling, including detailed assessment of 3-generation family history and histopathologic confirmation of DGC diagnoses in the family, to confirm HDGC status. Genetic testing for germline mutation in *CDH1* was initiated in affected probands, and testing of unaffected individuals was offered in 29 families. All genetic testing was performed in clinically accredited UK NHS laboratories and included analysis for large deletions as well as point mutations and indels. Patients in whom *CDH1*+ was detected were advised to undergo RRTG, but those choosing to delay surgery were enrolled in the endoscopic surveillance program. Individuals from families who were *CDH1*-NPVD were also offered endoscopic monitoring.

### Endoscopic protocol

Endoscopies were performed according to a standardized protocol previously described with 30 minutes for each procedure.^17^ Briefly, a white-light high-resolution endoscope with 85× magnification and a maximal resolution of 7.9 μm (GIF-FQ260Z; Olympus, Tokyo, Japan) was used to examine all anatomic segments of the insufflated stomach. Any abnormalities on white-light endoscopy were recorded and assessed further with autofluorescence imaging and narrow-band imaging magnification. Two highly experienced endoscopists (M.d.P and K.R.) conferred on any focal lesions; R.C.F. also had significant previous expertise in lesion recognition and reviewed the images as required.

Targeted biopsy specimens were taken from identified lesions, and 5 random biopsy specimens each were taken of the prepylorus, antrum, transitional zone, body, fundus, and cardia segments. Biopsy specimens were stained with hematoxylin and eosin and periodic acid–Schiff diastase and examined for the presence of SRCC foci by an upper specialist GI pathologist (M.O.), who had significant experience in SRCC identification. Any lesions were checked by a second pathologist within the Cambridge Pathology team before reporting. If required, we also consulted with F.C. based in Porto, who has many years unique experience in grading HDGC early lesions.

### Surveillance schedule

*CDH1*+ patients had endoscopies at 1-year intervals. Those who had SRCC detected and who chose to continue on endoscopic surveillance were offered 6-month endoscopies thereafter. *CDH1*-NPVD patients started with yearly endoscopies; after 2 negative procedures, the frequency was reduced to 2-year intervals.

### Data collection

Demographic information was collected and rapid urease test used to screen for *Helicobacter pylori* at baseline. Detection of SRCC foci on histopathology constituted an event, and the time to event from the first endoscopy was calculated. Follow-up time was defined as the time from first endoscopy to either the first endoscopy at which SRCC was detected or the most recent endoscopy (if no findings of SRCC).

Quality of life (QoL) was assessed at baseline and within 2 weeks of each endoscopy with European Organization for Research and Treatment of Cancer Quality of Life Questionnaire Core 30 (EORTC-QLQ-C30) version 3, a well-validated tool measuring QoL of cancer patients,^20^, ^21^ and 36-item Short Form Health Survey (SF-36) version 2 questionnaires, a generic tool for physical and mental health status.^22^ Functional scales of both were scored 0 to 100, with higher score representing better functioning. Patients who failed to complete their questionnaire after an endoscopy continued to be followed.

### Statistical analysis

Descriptive statistics were used to summarize cohort characteristics. Independent-sample Mann-Whitney U and χ^2^ tests identified differences in demographic characteristics and history of *H pylori* between the *CDH1*+ and *CDH1*-NPVD groups. Kaplan-Meier analysis with log rank test determined the difference in progression to SRCC on endoscopy according to *CDH1* mutation status, and Cox regression on *CDH1* status, controlling for all covariates (age unit 1, reference categories male [vs female], white [vs Asian], no proton pump inhibitor [PPI] use [vs PPI use], and no history of *H pylori* [vs *H pylori*]) was performed. Linear mixed models were used to determine significant differences between QoL scores pre-endoscopy and at each year of surveillance, controlling for age, gender, and *CDH1* status.

Level of significance was *P* ≤.05. Power analysis showed that a sample size of 13 in each of the *CDH1*+ and *CDH1*-NPVD groups (total of 26) was necessary to detect a difference in SRCC yield of 50%, to achieve a power of .9 (1-β), and α = .05. All statistical analyses were performed in SPSS version 23 for Windows (IBM Corp, Armonk, NY).

## Results

### Cohort characteristics

A total of 85 individuals from 46 families fulfilled HDGC criteria (Table 1); 54 (63.5%) had a pathogenic *CDH1* variant identified (Table 2). Of the remaining 31 patients (20 families) enrolled solely based on HDGC criteria, 11 tested negative for *CDH1* mutation, 13 were from families where no pathogenic variant had been identified in the affected index case, and 7 had unconfirmed mutation status. For 5 individuals there was no available DNA from affected family members for testing, and they were not offered testing because of limited confirmations of DGC in the family. For 2 the decision on whether to offer genetic testing had yet to be made, pending contact with other family members.

**Table 1:** Patient characteristics

	Overall (n = 85)	*CDH1*+ (n = 54)	*CDH1*-NVPD (n = 31)
Median age, y (IQR)	38 (28-50.5)	33.5 (26-46.3)	45 (32-57)
Sex, M:F	43:42	28:26	15:16
Ethnicity			
White	75	44	31
Asian	10	10	0
*H pylori* status, % positive test history	8.2	3.7	16.1
PPI use, % positive	27.1	25.9	29.0
Median follow-up time, mo (IQR)	12 (0-36)	0∗ (0-13)	34 (12-41)
Median number of endoscopies (IQR)	2 (1-3)	1 (1-3)	2 (2-3)
Findings of SRCC on biopsy specimen	36	33	3

*CDH1*+, *CDH1* pathogenic variant; *CDH1*-*NPVD*, *CDH1* no pathogenic variant detected; *IQR*, interquartile range; *PPI*, proton pump inhibitor.

∗ Follow-up time of 0 corresponds to 1 endoscopy.

**Table 2:** Pathogenic variants of the *CDH1* gene in *CDH1+* families

Family no.	*CDH1* pathogenic variant
1	exon 1 c.45-46insT, p.(Gln16fs)
2	deletion of exon 16
3	exon 10 c.1565+1G>T
4	exon 2 c.67C>T, p.(Gln23∗)
5	exon 10 c.1466-1467insC
6	deletion 828bp in exon 16
7	exon 5 c.641T>C, p.(Leu214Pro)
8	deletion exons 1 to 16
9	exon 10 c.1565+2dupT
10	deletion of exons 1 & 2
11	exon 7 c.1008+2T>G
12	exon 2 c.59G>A, p.(Trp20∗)
13	exon 12 c.1792C>T, p.(Arg598∗)
14	exon 5 c.639delG, p.(Trp213Cysfs∗2)
15	exon 10 c.1565+2dupT
16	deletion exons 1 to 16
17	exon 7 c.1008+2T>G
18	exon 7 c1003C>T, p.Arg335∗
19	exon 13 c.2164+2T>A
20	exon 2 c.49-1G>C
21	deletion of exon 3
22	exon 3 c.322delA, p.(Arg108Glufs∗9)
23	exon 3 c.385C>T, p.(Gln129∗)
24	exon 14 c.2248G>A, p.(Asp750Asn)
25	exon 12 c.1792C>T, p.(Arg598∗)
26	exon2 c.49-2A>G

*CDH1*+, *CDH1* pathogenic variant.

Two hundred one endoscopies were carried out, 118 on *CDH1*+ patients (median, 1 per patient; range, 1-9) and 83 on *CDH1*-NPVD patients (median, 2 per patient; range, 1-8). Overall, *CDH1*-NPVD patients underwent significantly more endoscopies (*P* = .015). The endoscopic schedule was subject to some variability depending on the personal availability and choice of patients. For *CDH1+* patients, the interval between endoscopies ranged from 8 to 15 months and for *CDH1*-NPVD, 1 to 3 years. One *CDH1*+ patient and 3 *CDH1*-NPVD patients discontinued from the program; all other participants reached the endpoint (detection of SRCC), proceeded to gastrectomy, or still remain in the program.

There was a significant difference in median age (33.5 vs 45, *P* = .010) and ethnicity (44:10 vs 31:0 white-to-Asian, *P* = .012) between the *CDH1*+ and *CDH1*-NPVD groups. However, there were no significant differences in terms of sex (*P* = .824), long-term PPI use (*P* = .803), or *H pylori* status (*P* = .094).

### Endoscopic findings

**Figure 1:**
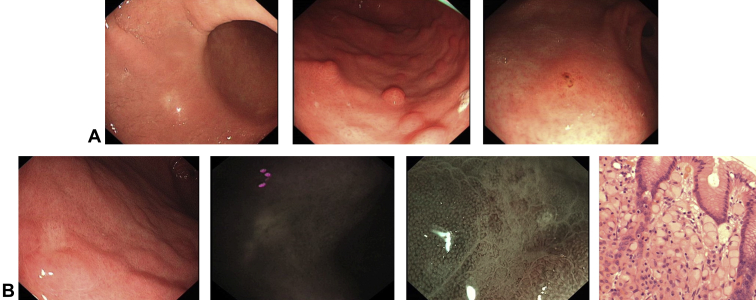
Detection of signet ring cell carcinoma (SRCC) on endoscopy. **A,** Examples of endoscopic lesions associated with foci of SRCC. From left to right: pale area, polyp, mucosal erosion. **B,** An additional case of a pale area positive for SRCC, with corresponding autofluorescence imaging and narrow-band imaging magnification image and histology showing a focus of SRCC. Narrow-band imaging magnification demonstrates loss of pit and irregular vessels.

Thirty random biopsy specimens were taken during each endoscopy. Gastric mucosal abnormalities were found in 124 of 201 endoscopies (62 patients), and 181 lesions, comprising 97 pale areas, 46 polyps, 27 erosions, and 11 nodular areas, were targeted for biopsy (Fig. 1, Table 3). Ten of the 181 targets showed autofluorescence imaging positivity, of which 1 contained SRCC foci, and 16 lesions had an irregular narrow-band imaging pattern, of which 5 were positive for SRCC. Of the 36 patients who had SRCC foci detected during endoscopic surveillance, 21 (58.3%) were diagnosed via random biopsy sampling only, 9 (25.0%) were diagnosed by targeted biopsy sampling only, and 6 (16.7%) had findings on both (Table 4). The locations in the stomach of SRCC foci in endoscopic biopsy specimens and gastrectomy specimens are shown in Table 5.

**Table 3:** Sensitivity, specificity, positive predictive value, and negative predictive value of targeted lesions

	Sensitivity (%)	Specificity (%)	Positive predictive value (%)	Negative predictive value (%)
Polyp	2.8	65.7	2.2	71.0
Pale areas	27.8	36.5	10.3	65.8
Erosions	11.1	81.3	14.8	75.8
Nodular	2.8	92.0	9.1	81.9

**Table 4:** Sensitivity, specificity, positive predictive value, and negative predictive value of endoscopy techniques

	Sensitivity (%)	Specificity (%)	Positive predictive value (%)	Negative predictive value (%)
Random	75.0	N/A	N/A	94.8
WLE targeted	41.7	N/A	N/A	83.1
AFI	2.8	91.4	10.0	73.1
NBI	13.9	89.3	31.3	74.8

*N/A*, not applicable; *WLE*, white-light endoscopy; *AFI*, autofluorescence imaging; *NBI*, narrow-band imaging.

**Table 5:** Locations of SRCC foci in endoscopic biopsy specimens and gastrectomy specimens

	Cardia	Fundus	Body	Antrum	Transitional zone	Total
Endoscopy	2	17	4	11	8	42
Gastrectomy	11	22	21	14	12	80

*SRCC*, Signet ring cell carcinoma.

### *CDH1+* patients

Of the 54 patients with *CDH1* mutation, 33 (61.1%) had foci of SRCC detected during surveillance. The interquartile range of follow-up time for these patients was 0 to 12 months, reflecting the high number of individuals who had lesions detected at their index endoscopy denoted time of 0. Twenty-two patients with endoscopic SRCC proceeded to RRTG. One patient who delayed gastrectomy at his own behest for 3.5 years despite identification of SRCC on the first endoscopy developed invasive poorly differentiated adenocarcinoma (pT4aN1M0), which was treated with radical gastrectomy with adjuvant chemotherapy. He remains well 4 years later. The remaining 10 patients had not committed to surgery at the time of writing. Of the 33 individuals with SRCC, 16 decided to undergo RRTG immediately. Although by definition follow-up ended at the first detection of SRCC, a significant proportion of patients reluctant to pursue surgery were continued on surveillance (with the same endoscopic protocol) to look for further lesions and monitor changes in SRCC morphology indicative of disease progression, with the current protocol at this center being endoscopy every 6 months. An additional 38 endoscopies were done in 14 patients after first detection of SRCC, and in 4 of these patients identification of atypia within the SRCC foci at subsequent endoscopy prompted clinicians to advise urgent gastrectomy. The remaining 3 patients had SRCC foci detected at their most recent endoscopy and have not had the opportunity to undergo either RRTG or further endoscopies at the time of writing.

For the 21 *CDH1*+ patients with no evidence of SRCC so far, the median follow-up was 8 months (interquartile range, 0-37), which differed significantly from those with SRCC (*P* = .035). Despite no findings, 6 of these patients proceeded to gastrectomy, all after the first endoscopy, and 4 were found to have SRCC upon examination of the surgical specimen. Reasons for their choice were concern about cancer risk, fellow siblings having surgery, and experience of recent diagnosis of invasive cancer or death in the family.

Although excluded from the primary analysis, a further 3 *CDH1*+ patients each had a single baseline endoscopy at which invasive adenocarcinoma was diagnosed. Two had clinical symptoms, including epigastric pain, nausea, and early satiety, in the 3 to 4 months prior but had not sought medical advice until after their genetic test result. As a result of endoscopy findings, a CT scan and laparoscopic staging was performed in all patients. In 2 patients, findings of metastatic disease precluded gastrectomy, and they were referred for palliative chemotherapy. The other patient (with pT4N1M0 diffuse-type poorly differentiated adenocarcinoma) proceeded to total gastrectomy with adjuvant chemotherapy.

### *CDH1-*NPVD patients

Foci of SRCC were detected in 3 of 31 patients (9.7%) from families where no *CDH1* pathogenic variant has been identified. Two were siblings from a family with strong history of DGC (1 parent and sister deceased) who had SRCC detected after 17 months of surveillance (on the second endoscopy); both underwent total gastrectomy 2 months later. Full *CDH1* genetic testing was carried out in both patients and affected relatives, but no pathogenic variants in *CDH1* have been found. The third patient tested negative for *CDH1* mutation before the start of surveillance and had a focus of SRCC detected on the third endoscopy (after 3 years of surveillance).

### Comparison of yield from endoscopy in *CDH1+* versus *CDH1-*NPVD patients

**Figure 2:**
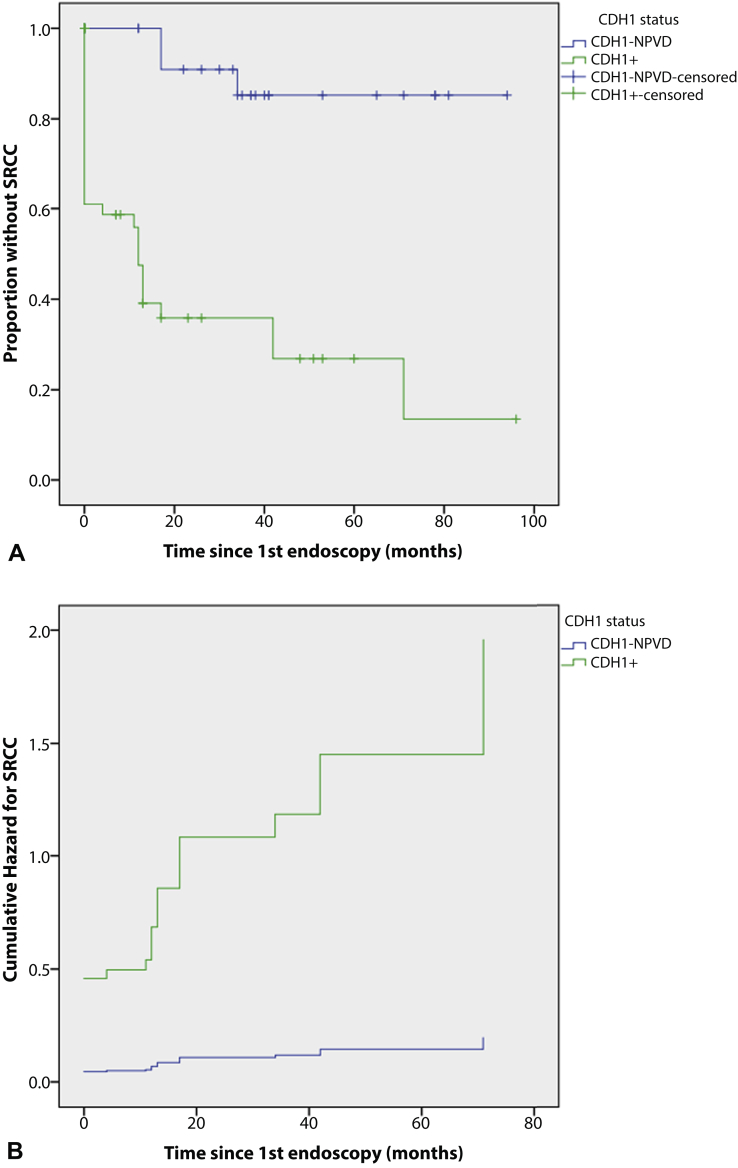
Risk of signet ring cell carcinoma (SRCC) on endoscopy by *CDH1* mutation status. **A,** Survival analysis of progression to SRCC on endoscopy by *CDH1* mutation status. **B,** Cumulative hazard for SRCC by *CDH1* mutation status. *CDH1*-*NPVD*, *CDH1* no pathogenic variant detected.

Kaplan-Meier analysis showed that *CDH1*+ patients had significantly higher progression to endoscopic detection of SRCC than *CDH1*-NPVD patients (*P* < .0005) (Fig. 2A). *CDH1*+ patients had a 10-fold risk of SRCC on endoscopy relative to *CDH1*-NPVD patients (hazard ratio, 10.06; 95% confidence interval, 2.92-34.68; *P* < .0005), whereas no other factors, including age (*P* = .939), sex (*P* = .762), ethnicity (*P* = .769), PPI use (*P* = .860), and *H pylori* status (*P* = .844), were associated with risk of SRCC (Fig. 2B).

### Time course of findings of SRCC on endoscopy in *CDH1*+ patients

**Figure 3:**
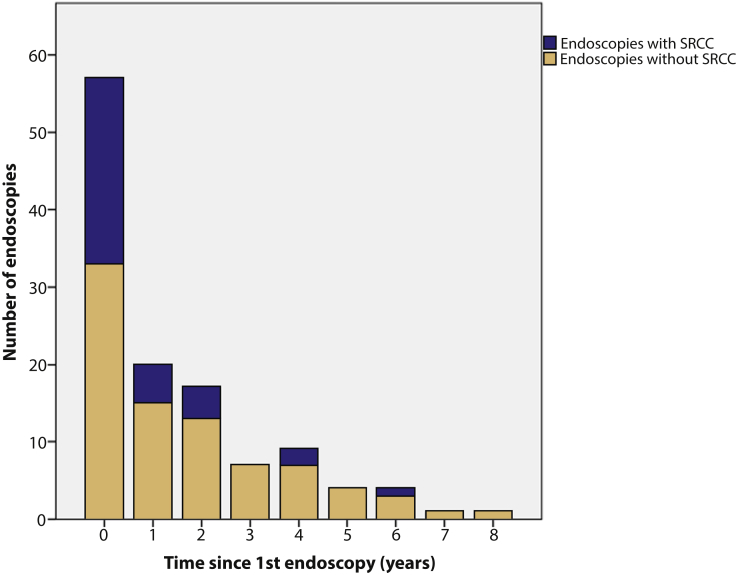
Timing of findings of signet ring cell carcinoma on endoscopy in *CDH1*+ patients.

In *CDH1*+ patients, most findings of SRCC were made at the first endoscopy (21/33, 63.6%), at which the likelihood of positive findings was 38.9% (21/54). The detection rate fell to 23.8% (5/21) in the 12 months after the first endoscopy and 23.5% (4/17) in the second year of surveillance. After the fourth year, only 1 finding of SRCC was made (in 10 endoscopies) (Fig. 3). For findings after the first endoscopy, median time from first endoscopy to detection of SRCC was 13 months, at which point 87.9% of findings had been made.

### QoL of *CDH1+* and *CDH1-*NPVD patients undergoing endoscopy

Sixty-eight sets of EORTC-QLQ-C30 and SF-36 questionnaire data were obtained from 47 patients (33 *CDH1*+, 14 *CDH1*-NPVD), and outcomes for the 8 domains of the SF-36 and QoL domain and 5 functional scales of the EORTC-QLQ-C30 were calculated. There appeared to be a trend toward improved QoL and psychological health with time on the surveillance program. Mean scores for QoL (*P* = .009) and emotional function (*P* = .042) on the EORTC-QLQ-C30 and mental health on the SF-36 (*P* = .014) were significantly improved at 5 years compared with pre-endoscopy baseline (Table 6). There were no significant differences in any other domain.

**Table 6:** Quality of life measures by time on the endoscopic surveillance program

	Pre-endoscopy (n = 10)	First endoscopy (n = 23)	1 year (n = 10)	2 year (n = 7)	3 year (n = 7)	4 year (n = 4)	5 year (n = 4)
EORTC-QLQ-C30: QoL	73.3 (16.1)	76.1 (21.1)	76.7 (21.8)	77.8 (10.1)	76.2 (26.5)	64.6 (18.5)	93.8 (8.0)
EORTC-QLQ-C30: Phys F	94.5 (10.8)	96.8 (6.3)	100	96.7 (3.7)	100	96.7 (3.8)	98.3 (3.3)
EORTC-QLQ-C30: Role F	93.3 (11.7)	95.7 (11.5)	91.7 (11.8)	91.7 (20.4)	83.3 (37.3)	91.7 (16.7)	100
EORTC-QLQ-C30: Emot F	66.7 (31.9)	78.6 (22.7)	73.3 (22.2)	83.3 (10.5)	85.7 (21.9)	77.1 (21.9)	93.8 (12.5)
EORTC-QLQ-C30: Cog F	80.0 (29.2)	89.1 (17.8)	90.0 (16.1)	94.4 (13.6)	90.5 (13.1)	91.7 (16.7)	87.5 (16.0)
EORTC-QLQ-C30: Soc F	90.0 (14.1)	93.5 (12.0)	91.7 (14.2)	97.2 (6.8)	95.2 (12.6)	100	100
SF-36: Phys F	97.5 (6.3)	95.1 (8.8)	96.7 (4.3)	90.7 (14.6)	98.3 (2.6)	100	98.8 (2.5)
SF-36: Role PF	91.9 (13.8)	92.1 (14.0)	94.4 (14.5)	78.6 (29.1)	89.6 (20.0)	95.3 (9.4)	98.4 (3.1)
SF-36: Bod Pain	77.6 (24.0)	81.5 (17.6)	85.1 (15.2)	90.0 (14.5)	81.2 (24.7)	100	96.0 (8.0)
SF-36: Gen Hlth	54.2 (7.2)	55.0 (7.1)	58.9 (10.0)	53.9 (14.0)	56.0 (6.4)	59.0 (15.7)	59.3 (1.5)
SF-36: Vitality	57.5 (25.5)	62.2 (24.9)	62.5 (30.8)	55.4 (22.9)	59.4 (29.2)	57.8 (23.6)	75.0 (10.2)
SF-36: Soc F	77.5 (26.9)	81.0 (23.8)	81.9 (29.4)	80.4 (30.5)	85.4 (22.9)	93.8 (7.2)	100
SF-36: Role EF	81.7 (23.5)	87.3 (20.7)	85.2 (21.6)	65.5 (40.7)	91.7 (12.9)	89.6 (12.5)	100
SF-36: Men Hlth	66.5 (16.7)	67.4 (20.5)	71.7 (19.4)	68.6 (18.9)	75.8 (21.3)	77.5 (9.6)	88.8 (10.3)

Values are means with standard deviation in parentheses. *EORTC-QLQ-C30*, European Organization for Research and Treatment of Cancer Quality of Life Questionnaire Core 30; *SF-36*, 36-item Short Form Health Survey; *QoL*, quality of life/global health status; *Phys F*, physical functioning; *Role F*, role functioning; *Emot F*, emotional functioning; *Cog F*, cognitive functioning; *Soc F*, social functioning; *Role PF*, role limitation because of physical health; *Bod Pain*, bodily pain; *Gen Hlth*, general health; *Role EF*, role limitation because of emotional problems; *Men Hlth*, mental health.

## Discussion

In this prospective cohort study of endoscopic surveillance in HDGC families, the overall yield of SRCC foci was 36 of 85 (42.4%). Yield was significantly greater in those with a *CDH1* mutation (61.1% compared with 9.7% in *CDH1*-NPVD patients, *P* < .0005).

### Endoscopy in *CDH1+* patients

The SRCC detection rate in *CDH1*+ patients was similar to our previous report (63.6%), which shows yield is maintained in a larger cohort.^17^ Our results represent a substantial improvement in yield compared with other studies that have reported detection rates of 9% to 16%.^23^, ^24^, ^25^ In a series of 93 chromoendoscopies, 10 of 33 *CDH1*+ patients (30.3%) had SRCC identified.^19^ We did not use chromoendoscopy but did use autofluorescence imaging and narrow-band imaging, although it is unclear whether these improve detection compared with white light.^17^ The superior yield in our study is likely because of the use of an optimized and standardized endoscopic protocol, performed on a dedicated endoscopy list by the same team of endoscopists. It may also be relevant that our protocol included random biopsy sampling, in contrast to Shaw et al,^19^ who only took targeted biopsy specimens of pale areas. Random biopsy sampling had a 75% sensitivity for the detection of SRCC, whereas the sensitivity of targeted biopsy sampling was 41.7%. Thus, both random and targeted biopsy sampling have a place in endoscopic surveillance.

The importance of a baseline endoscopy in *CDH1*+ individuals is underscored by the fact that 3 individuals had invasive cancer detected. These patients would not have been served well by proceeding directly to gastrectomy before formal staging.

Observations in surgical specimens from asymptomatic *CDH1*+ individuals suggest that the penetrance for development of SRCC foci is virtually 100%,^9^, ^26^, ^27^, ^28^, ^29^ indicating that there are likely patients who harbor SRCC who have not yet been diagnosed in this study because of the microscopic nature of foci, often lying beneath intact epithelium, that may be missed on biopsy sampling.^30^ Therefore, caution is necessary when delaying gastrectomy when no SRCC are found. However, most SRCC foci in *CDH1*+ stomachs, limited to the gastric mucosa, are small (<1 mm diameter) and contain signet ring cells that are mitotically inactive. Progression to advanced HDGC is associated with development of larger SRCC foci (>3 mm) containing increased numbers of poorly differentiated cancer cells, which are highly proliferative and invade the muscularis mucosae and muscularis propria. Endoscopic surveillance has increased the chance of detecting larger foci and hence is of value for identifying SRCC with risk of cancer progression and advising on the need for surgery.^31^, ^32^, ^33^ In our experience, given the magnitude of the decision for surgery at a young age and in some cases in which there are medical contraindications to surgery, many patients choose endoscopy, which can be useful for ruling out invasive disease and help patients to reach a decision and guide them on timing of surgery.

### Reassessing the role of endoscopy in *CDH1-*NPVD families

In contrast to *CDH1*+, there was a very small yield of SRCC in individuals from *CDH1*-NPVD families despite significantly longer follow-up. The 9.7% yield here represents a substantial decrease from our previous finding of 28.6%.^17^ This current estimate is likely to be more accurate because of the significant enlargement of the *CDH1*-NPVD cohort in this study (31 patients compared with the previous 7 patients). These results suggest that endoscopic screening to detect SRCC is of limited value in *CDH1*-NPVD patients. This has significant clinical implications given that most families fulfilling HDGC criteria have no confirmed *CDH1* mutation, and as genetic cancer services are expanded, particularly in high GC incidence countries, the burden on healthcare resources of surveillance for *CDH1*-NPVD families will increase.

There are several possible reasons for the lack of SRCC detection in *CDH1*-NPVD patients. In families with an autosomal dominant pattern of inheritance, at least half of individuals undergoing endoscopy are likely to be wild-type and so are not at risk. It is also possible that SRCC are not the precursor lesions for some pathways to HDGC. Van der Post et al^6^ reviewed the gastric specimens of 103 HDGC families without *CDH1* mutations, and although advanced cancers showed similar morphology to that of mutation carriers, in situ SRCC and pagetoid spread of signet ring cells were not present. However, in another study, 4 members of a family with a germline truncating *CTNNA1* mutation had intramucosal signet ring cells detected on endoscopic biopsy specimens.^13^ More work is required to elucidate the pathogenesis of *CDH1*-NPVD tumors to allow the establishment of more reliable markers for screening.

In the absence of reliable surveillance methods, management of *CDH1*-NPVD families should focus on risk stratification, including the search for other germline genetic variants and clinical and environmental risk factors. Pathogenicity of variants should be clarified using a combination of methods.^34^, ^35^, ^36^, ^37^ *CDH1* allelic expression imbalance (AEI) may be a key factor. A study found, expectedly, a high AEI in a large proportion (80%) of *CDH1*+ patients, but high AEI was also seen in many (12/17, 70%) *CDH1*-NPVD patients. Importantly, AEI was not seen in any of the 21 patients without cancer, indicating that it may be a good marker for cancer development. AEI may increase the proportion of HDGC cases linked to *CDH1* abnormalities to 80%.^38^ Hence, incorporation of *CDH1* AEI assessment into the workup of mutation-negative families could be considered. The use of whole exome sequencing and unbiased next-generation sequencing technologies will facilitate the discovery of further HDGC-associated genes, which will hopefully allow the genetic cause to be identified in more families lacking *CDH1* mutations.

### Endoscopy yield over time and implications for scheduling

Most findings of SRCC were made on the first endoscopy, and there was a sharp drop in the number of findings over time (although the numbers were small). The likely explanation is that the protocol detected prevalent rather than incident cases, with some foci not detected on the first endoscopy because of the imperfect sensitivity of endoscopy. This is consistent with the previous literature on HDGC pathogenesis, which suggests that multiple foci of SRCC develop in *CDH1* mutation carriers before the age of 30.^5^, ^9^, ^39^, ^40^ The *CDH1*+ patients in this study (median age, 33.5 years) are likely to have already developed SRCC before starting surveillance. Given that the average age of the *CDH1*-NPVD cohort was 45 years, this further reinforces the hypothesis that such individuals are not at high risk of classic HDGC.

Our observations differ from that of a chromoendoscopy surveillance program, in which 4 of 10 patients with SRCC detected were diagnosed at or after the fifth endoscopy and the number of patients diagnosed per procedure doubled each year.^19^ Shaw et al^19^ attributed this to a learning curve effect, with improved detection of smaller pale areas and accuracy of biopsy sampling with experience. These factors would not have affected our study because we used a standardized protocol and endoscopists had significant previous expertise in lesion recognition. These results suggest that frequency of endoscopy could be reduced over time, and a formal protocol could be developed of, for example, annual endoscopy for 4 years, reduction to endoscopy every 2 years after 4 negative procedures, and referral back to local team after 10 years if all results are negative.

### Psychological health during endoscopic surveillance

We found that the QoL and mental health of participants improved with time on the surveillance program. This is likely because of the reassurance that patients derive from having monitoring and psychological and emotional benefit gained from support from the research nurse and other members of the multidisciplinary team, including continued counseling. This is interesting because a recent study by Hallowell et al^18^ suggested that difficulty tolerating repeated endoscopies is a factor influencing the decision to undergo gastrectomy in *CDH1*+ patients. The discrepancy may be because of methodologic differences: Hallowell et al conducted qualitative interviews, whereas we used standard questionnaires, which asked patients about their general feelings and experiences rather than questions specifically related to endoscopy. Our findings suggest the value of an intensive service for patient well-being and perhaps the need, if patients have less-frequent endoscopy or are discharged from the program, for maintained provision of support and counseling through alternative channels.

This study is not without limitations. The sample size was relatively small, although for this rare condition it is larger than most other reported studies to date. The study was focused on the yield of the program rather than the cost-effectiveness. QoL data were only collected from 46 of 85 patients and only on a small number of individuals beyond the 3-year time point, so there is a risk of bias.

### Conclusions

This study confirms the high prevalence of SRCC foci in patients with a germline *CDH1* mutation. A baseline endoscopy should be performed in all *CDH1*+ patients to exclude invasive disease. Most patients should be advised to have an RRTG, but where gastrectomy is not the preferred option, identification of SRCC foci is common by the fourth endoscopy and can help inform patients and physicians about the recommended timing of surgery. For *CDH1*-NPVD patients, endoscopy can provide the only definitive evidence of their risk status. However, the yield is low, and the value of continued monitoring for patient well-being needs to be balanced against the resource burden. In the future, it is envisaged that more germline risk variants will be identified to help better manage these families.
